# Influence of game and quarter results on external peak demands during games in under-18 years, male basketball players

**DOI:** 10.5114/biolsport.2023.116010

**Published:** 2022-06-01

**Authors:** Enrique Alonso Pérez-Chao, Miguel Ángel Gómez, Aaron Scanlan, Carlos Ribas, Juan Trapero, Alberto Lorenzo

**Affiliations:** 1 Facultad de ciencias de la actividad física y del deporte, Universidad Politécnica de Madrid, 28040 Madrid, Comunidad de Madrid, Spain; 2 Faculty of Sports Sciences, University Alfonso X el Sabio, 28691 Villanueva de la Cañada, Community of Madrid, Spain; 3 Human Exercise and Training Laboratory, School of Health, Medical and Applied Sciences, Central Queensland University, Rockhampton, Queensland, Australia; 4 School of Sports Science, European University of Madrid, 28670 Villaviciosa de Odón, Spain, Villanueva de la Cañada, Community of Madrid, Spain

**Keywords:** Team sports, Worst case scenario, Most demanding scenarios, Local positioning system, Load monitoring, Game demands

## Abstract

To quantify and compare the external peak demands (PD) encountered according to game result (win vs. loss), quarter result (win vs. tie vs. loss), and quarter point difference (± difference in score) in under-18 years (U18), male basketball players. Thirteen basketball players had external load variables monitored across 9 games using local positioning system technology, including distance covered, distance covered in different intensity zones, accelerations, decelerations, and PlayerLoad™. PD were calculated across 30-s, 1-min, and 5-min time windows for each variable. Linear mixed models were used to compare PD for each variable according to game result (win vs. loss), quarter result (win vs tie vs loss), and quarter point difference (high vs. low). External PD were comparable between games that were won and lost for all variables and between quarters that were won and lost for most variables (p > 0.05, trivial-small effects). In contrast, players produced higher (p < 0.05, small effects) 1-min high-speed running distance and 5-min PlayerLoad^TM^ in quarters that were won compared to quarters that were lost. Additionally, high quarter point differences (7.51 ± 3.75 points) elicited greater (p < 0.05, small effects) external PD (30-s PlayerLoad^TM^, 30-s and 5-min decelerations, and 1-min and 5-min high-speed running distance) than low quarter point differences (-2.47 ± 2.67 points). External PD remain consistent (trivial-small effects) regardless of game result, quarter result, and quarter point difference in U18, male basketball players. Accordingly, external PD attained during games may not be a key indicator of team success.

## INTRODUCTION

Recent technological advances such as electronic performance tracking systems (EPTS), including inertial measurement units (e.g. accelerometers and gyroscopes) (IMU) [1, 2] and local positioning systems (LPS) [3] have allowed for basketball players to be monitored during training and games. There are several advantages to using this technology, including the capability to quantify the external loads of several players simultaneously to gather monitoring data efficiently in real time [4]. In this regard, external load is regarded as the physical load performed (e.g., duration, distance), which is determined by the organization, intensity, and quantity of exercise [5].

It is essential to quantify the external loads experienced during games among various samples of basketball players to develop training and recovery plans that are specific to player sex, competition level, and age. Regarding player age, quantifying external game loads among junior basketball players (< 18 years of age [U18]) is of particular interest given the importance of understanding the ratio of competition-to-training demands in optimizing the overall loading placed on young athletes across their development pathway [6]. In this way, external load data reported for U18, international, male basketball players (16.9 ± 1.1 years) show that basketball is an acyclic, high-intensity sport where playing time is mostly (93%) spent standing, walking (< 7 km · h^−1^), or jogging (7–14 km · h^−1^) [3]. These low-intensity movements are interspersed with high-intensity efforts [7, 8], during which players attain their peak demands (PD) [9–11]. Quantifying the external PD encountered by U18 basketball players during games is particularly important given the intensities detected can guide optimal training prescription to best prepare players to cope with most demanding scenarios experienced during games [12] and to assist these young players in transitioning to adult competitions. External PD are considered the most intense activity periods experienced by basketball players for a selected variable across a specified timeframe of interest [11, 12].

Theoretically, the ability to achieve higher external PD during games could indicate players may be capable of attaining a superior playing pace at key stages across games. In turn, superior playing pace may indicate an ability to outplay opponents from a physical perspective and improve the likelihood of making successful plays, contributing to team success [13]. Alternatively, higher external PD during games may be reflective of game contexts in which a team, that is losing at the time, might elevate playing intensity to improve the score-line. However, it is currently unknown whether the external PD reached during games differentiate team success. Nevertheless, recent evidence exploring the average external intensities completed across entire games demonstrated that players in the bottom four teams completed significantly (p < 0.05) higher relative distances (m · min^−1^) than players in the top four teams during games in an U18, European male competition [14]. Similarly, separate research quantifying the average external intensities attained across entire games in adult, semi-professional, male basketball players showed players executed significantly (p < 0.001) more high-intensity accelerations per minute during games that were lost compared to won [15]. In turn, research examining external PD during games in adult, semi-professional, male basketball players showed consistent (p > 0.05, trivial-small effects) relative PlayerLoad (AU · min^−1^) across 15-s, 30-s, 1-min, 2-min, 3-min, 4-min, and 5-min sampling durations (i.e., peak intensities averaged across different time windows) were achieved between quarters that were won and lost [13]. Consequently, the existing research suggests losing teams likely experience greater average external intensities across games [14, 15], but the intensity of the most demanding game passages are comparable across quarters that are won and lost [13].

However, it should be noted that only one study [13] has quantified and compared external PD between quarters that were won and lost in basketball players. Furthermore, this previous study [13] used PlayerLoad^TM^ as the only variable to quantify external PD, did not consider score-line margins, and examined adult basketball players. Further insight is needed regarding the PD encountered during basketball games according to team success using a wider selection of external load variables and specifically analyzing score-line margins. In this sense, game score-line has been shown to impact coaching substitution strategies [16] and therefore the active playing time of players, as well as team performance evidenced through improved score-line margins across game quarters when in a losing position at the start of the quarter [17]. Furthermore, separate research on this topic is needed in U18 basketball players for the generation of age-specific evidence given U18, male basketball players (16.8 ± 0.6 years) have been shown to cover more total distance (p < 0.05, moderate effect) and highspeed running distance > 18 km · h^−1^ (p < 0.05, small effect) across 1-min time windows than adult, male basketball players (19.6 ± 1.5 years) within a Euroleague academy setting [18].

Research ascertaining whether external PD differentiate team success across games and individual quarters considering the score-line margin will provide evidence for basketball practitioners to formulate training strategies to optimize player readiness for games and likelihood of team success. Therefore, the aim of this study was to quantify and compare the PD encountered according to game result (win vs. loss), quarter result (win vs. tie vs. loss), and quarter point difference (± difference in scores) across different time windows and external load variables in U18, male basketball players. Based on existing data [13, 14], it was hypothesized that losing teams would experience greater external PD across games and in individual quarters.

## MATERIALS AND METHODS

### Sample

Male basketball players from the same team competing in the highest regional division of an U18 Spanish basketball competition (n = 13, mean ± standard deviation [SD]: age 16.6 ± 1.0 years, height 197.6 ± 8.0 cm, body mass: 87.8 ± 7.7 kg) were monitored during nine official games played in the same stadium. For inclusion in the study, players had to complete at least 15 min of playing time in at least five of the nine games. Furthermore, to ensure adequate time windows for external PD variables could be calculated in each quarter, data from players who did not complete at least 5 min of playing time in a specific quarter were removed from individual quarter analyses but not entire game analyses. Playing time was classified as the time (min) each player was on the court during games, including stoppages (i.e., free-throws, fouls, out-of-bounds, rule infringements) but excluding warm-up, breaks between quarters, and timeouts. Consequently, three players originally recruited for the study did not meet the inclusion criteria and were excluded from final analyses, resulting in 13 players being retained in the study. Considering entire game analyses, 117 game samples were included in final analyses. Considering individual quarter analyses, 198 quarter samples were excluded due to players not attaining at least 5 min of playing time, resulting in 270 quarters samples across the 13 players remaining in final analyses. This study was conducted in accordance to the Declaration of Helsinki [19] and approved by the Institutional Review Board of the Polytechnic University of Madrid, Spain.

### Procedures

This observational investigation was conducted across a 5-month period throughout the 2019–2020 season. Each player wore a device (Vector S7; Catapult Sports, Melbourne, Australia) in a bespoke pocket within a vest positioned on the upper thoracic spine between the scapulae. The devices contained an accelerometer (± 16 g, 100 Hz), magnetometer (± 4,900 µT, 100 Hz), gyroscope (up to 2,000 deg/ sec, 100 Hz), and LPS. The ClearSky LPS (ClearSky S7, 10 Hz, firmware version 5.6.; Catapult Sports, Melbourne, Australia) is an ultra-wide band, 4-GHz transmitting system equipped with 24 anchors positioned around the perimeter of basketball stadium that was used to collect LPS data. The technology used in this study has been supported as valid in measuring distance [20–23], speed, accelerations, decelerations [20, 21], and Player Load^TM^ [24], while similar LPS technology has been shown to be reliable (coefficient of variation (CV) < 5%) in measuring distance and speed variables [23]. All players were familiarized with the monitoring technology, having worn the devices during training and games in the previous season. Each device was turned on ~20–40 min before the warm-up preceding each game. Players wore the same device throughout the study period to avoid inter-device variation in external load data outputs [25, 26].

To determine the external PD, first, the raw data was extracted in 1-s intervals for each player. Data were then exported to a custombuilt Microsoft Excel (version 16.0; Microsoft Corporation, Redmond, WA) spreadsheet for further analysis. Data were analyzed across different time windows (30 s, 1 min, and 5 min) using rolling averages to find the peak value for each variable across each duration. This method is commonly applied when determining external PD [27] and has been previously used in several basketball studies [10, 11, 18, 28]. Data were analyzed from the beginning of each quarter to the end of the same quarter, with data across all quarters in the same game collated together for game analyses (i.e., the highest value for each variable across any quarter in a specific game was taken as the PD for that game).

### Variables

PD were calculated for several distance variables including total distance (m) covered (TD) and distance (m) covered in different intensity zones including: standing-walking (S-W) ≤ 7 km · h^−1^; jogging (JOG) = 7–14 km · h^−1^’; running (RUN) = 14.01–18 km · h^−1^; and high-speed running (HSR) > 18 km · h^−1^, as previously used in basketball research [3]. Furthermore, accelerations (ACC) (count) performed > 2 m · s^−2^ (dwell time: 0.3 seconds), decelerations (DEC) (count) performed > -2 m · s^−2^ (dwell time: 0.3 s), and PlayerLoad™ (PL) (arbitrary units [AU]) were also measured. These dwell times were chosen given values between 0.3 and 0.4 s have been identified as the most readily used in basketball settings [10, 11, 29].

PL was calculated as the square root of the sum of the instantaneous rate of change in acceleration in the three movement planes (x-, y, and z-axis) using the following formula [11, 12]:

$$\begin{array}{c} PlayerLoad^{™}=[\sqrt{{\left(a_{y1}-a_{y-1}\right)}^{2}}\\ +\surd (a_{x1}-a_{x-1}{)}^{2}+\surd (a_{z1}-\alpha_{z-1}{)}^{2}]/100 \end{array}$$

where fwd indicates movement in the anterior-posterior direction, side indicates movement in the medial-lateral direction, up indicates vertical movement, and t represents time.

Comparisons according to game result were made between games that were won (8 games, 244 samples) and games that were lost (1 game, 26 samples). Comparisons according to quarter result were made between quarters that were won (24 quarters, 183 samples), quarters that were tied (2 quarters, 14 samples), and quarters that were lost (9 quarters, 73 samples). Additionally, using a two-step cluster analysis for quarter point difference (± difference in score), the sample was split into two groups (average silhouette = 0.7) as shown in Table 1.

**TABLE 1:** Cluster analysis identifying groups based on quarter point difference during games.

Measure	Low difference	High difference
Quarter point difference	-2.47 ± 2.67	7.51 ± 3.75
Sample size (N)	94	176
Proportion of samples (%)	34.8%	65.2%
Bayesian information criterion	168.47	

*Note*: Quarter point difference groups presented as mean ± standard deviation for each group; sample size indicates the number of individual quarter samples included across all players.

### Statistical analysis

The mean, standard deviation (SD), and coefficient of variation (CV) were determined for each PD variable across each time window and according to each factor (game result, quarter result, and quarter point difference). Linear mixed models (LMM) were used to compare each PD variable according to game result (i.e., win vs. loss), quarter result (win vs tie vs loss), and quarter point difference (i.e., high vs. low) across each time window. In each model, game result, quarter results, or quarter point difference was entered as the fixed term, and participant number was entered as the random term. Levene’s equality of variances test was performed to assess for equal variances in the data. Cohen’s effect size (ES) and the mean difference with 95% confidence intervals (CI) were determined for all pairwise comparisons and interpreted as: trivial ≤ 0.20; small = 0.20–0.59; moderate = 0.60–1.19; large = 1.20–1.99; and very large ≥ 2.00 [30]. All analyses were conducted using IBM SPSS for Windows (version 23, IBM Corporation, Armonk, New York), except ES, which were calculated using a customized Microsoft Excel spreadsheet (version 16.0, Microsoft Corporation, Redmond, WA).

## RESULTS

**FIG. 1:**
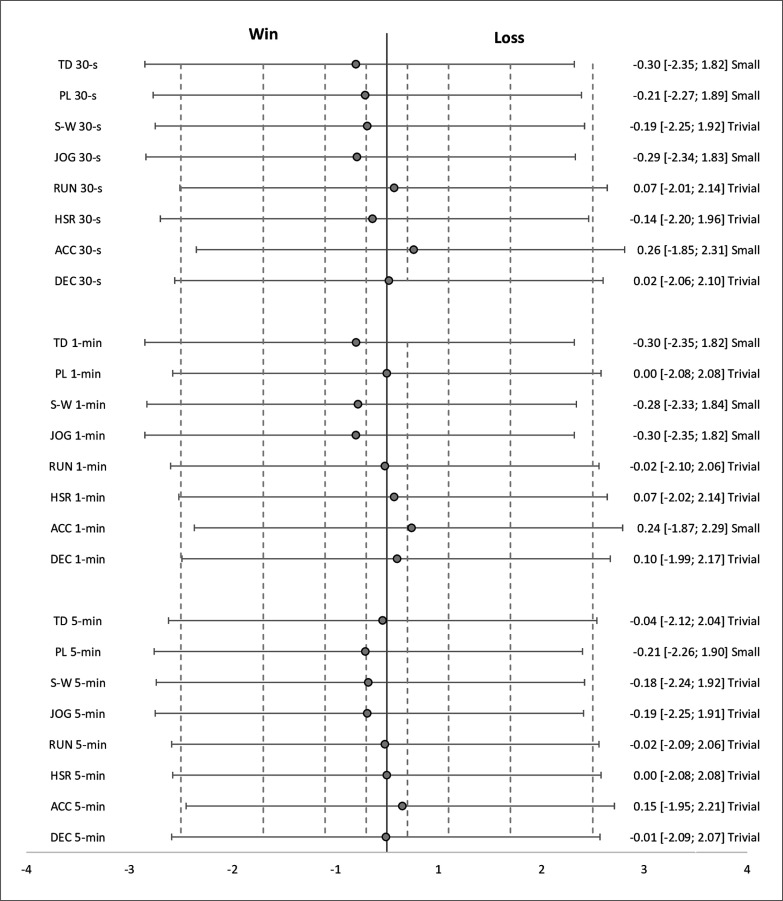
Effect sizes with 95% confidence intervals for comparisons in external peak demand variables between games that were won and lost. *Abbreviations:* TD = total distance, PL = PlayerLoad, S-W = standing-walking, JOG = jogging, RUN = running, HSR = highspeed running, ACC = accelerations, DEC = decelerations. *Note: Dotted lines represent ES interpretation (trivial* ≤ *0.20; small = 0.20– 0.59; moderate = 0.60–1.19; large = 1.20–1.99 and very large* ≥ *2.00).*

**FIG. 2:**
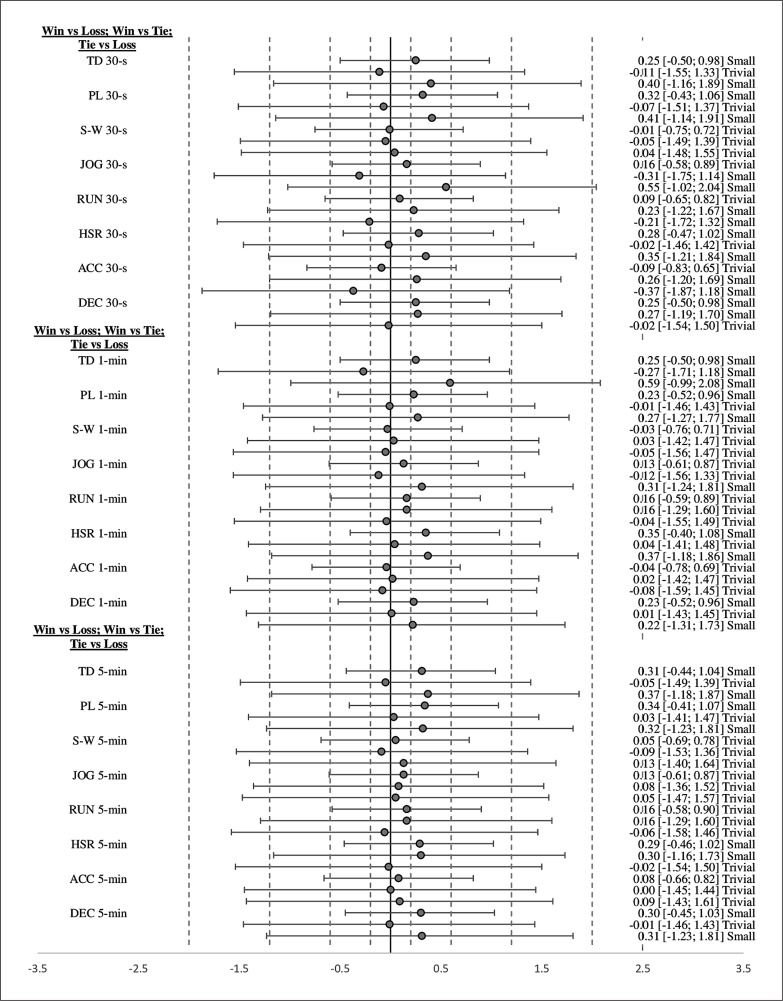
Effect sizes with 95% confidence intervals for comparisons in external peak demand variables between quarters that were won and lost. *Abbreviations:* TD = total distance, PL = PlayerLoad, S-W = standing-walking, JOG = jogging, RUN = running, HSR = high-speed running, ACC = accelerations, DEC = decelerations. *Notes: Dotted lines represent ES interpretation (trivial* ≤ *0.20; small = 0.20–0.59; moderate = 0.60–1.19 and large = 1.20–1.99). For each parameter the comparison followed is win vs loss; win vs tie; tie vs loss.*

**FIG. 3:**
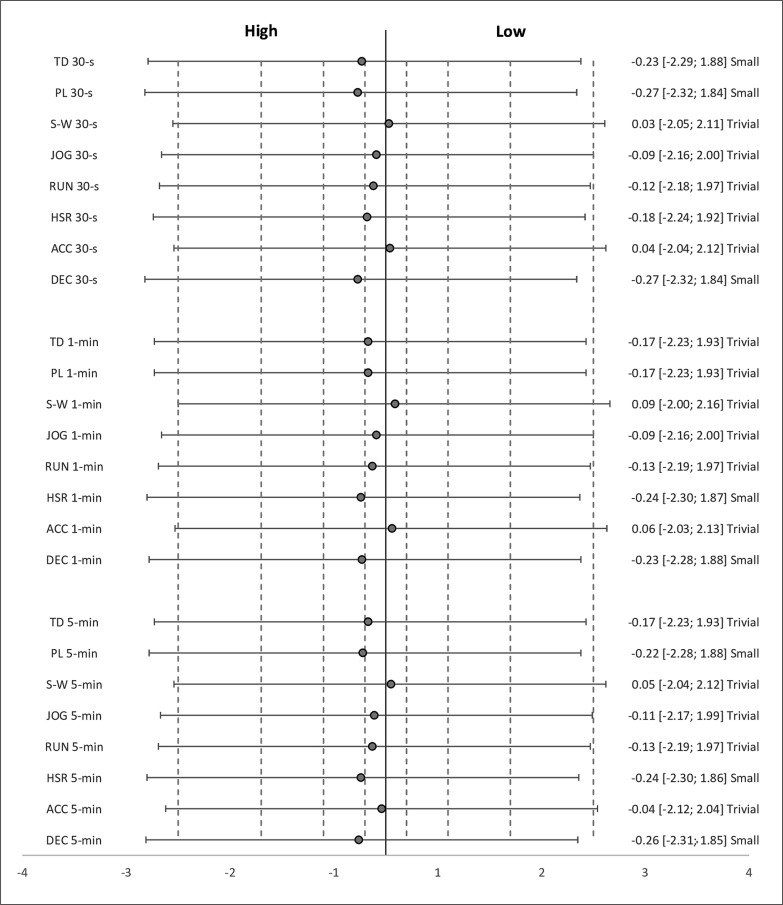
Effect sizes with 95% confidence intervals for comparisons in external peak demand variables between quarters with low (-2.47 ± 2.67 points) and high (7.51 ± 3.75 points) score-line margins. *Abbreviations:* TD = total distance, PL = PlayerLoad, S-W = standing-walking, JOG = jogging, RUN = running, HSR = high-speed running, ACC = accelerations, DEC = decelerations. *Note: Dotted lines represent ES interpretation (trivial* ≤ *0.20; small = 0.20–0.59; moderate = 0.60–1.19; large = 1.20–1.99 and very large* ≥ *2.00).*

Descriptive statistics for each variable across each time window according to each factor are presented in Tables 2–4. The estimated effects (ES ± 95% CI) for differences in pairwise comparisons according to each factor are presented in Figures 1–3.

**TABLE 2:** Descriptive statistics for external peak demand variables according to game result across different time windows.

30-s time window				1-min time window				5-min time window			

*Variable*	*Result*	*Mean ± SD*	*%CV*	*Variable*	*Result*	*Mean ± SD*	*%CV*	*Variable*	*Result*	*Mean ± SD*	*%CV*
**TD (m)**	*Win*	80.2 ± 8.7	10.8	**TD (m)**	*Win*	133.7 ± 14.6	10.9	**TD (m)**	*Win*	449.7 ± 69.4	15.4
	*Loss*	77.5 ± 7.6	9.8		*Loss*	129.3 ± 14.2	10.9		*Loss*	446.6 ± 66.2	14.8

**PL (AU)**	*Win*	10.4 ± 1.5	14.6	**PL (AU)**	*Win*	17.0 ± 2.6	15.7	**PL (AU)**	*Win*	53.3 ± 10.5	19.7
	*Loss*	10.1 ± 1.7	17.1		*Loss*	17.0 ± 3.0	18.1		*Loss*	51.1 ± 13.3	26.0

**S-W (m)**	*Win*	33.2 ± 6.3	18.9	**S-W (m)**	*Win*	57.5 ± 14.5	25.3	**S-W (m)**	*Win*	217.3 ± 69.7	32.0
	*Loss*	32.0 ± 4.5	14.1		*Loss*	53.5 ± 8.4	15.7		*Loss*	204.8 ± 52.6	25.7

**JOG (m)**	*Win*	44.5 ± 9.8	22.1	**JOG (m)**	*Win*	70.4 ± 20.6	29.2	**JOG (m)**	*Win*	200.2 ± 106.5	53.2
	*Loss*	41.7 ± 5.9	14.2		*Loss*	64.2 ± 9.6	14.9		*Loss*	180.0 ± 35.5	19.7

**RUN (m)**	*Win*	26.2 ± 9.2	35.2	**RUN (m)**	*Win*	35.4 ± 16.8	47.6	**RUN (m)**	*Win*	79.1 ± 74.0	93.6
	*Loss*	26.9 ± 5.3	19.8		*Loss*	35.0 ± 8.9	25.4		*Loss*	77.8 ± 19.3	24.9

**HSR (m)**	*Win*	17.2 ± 8.5	49.2	**HSR (m)**	*Win*	19.9 ± 10.3	51.9	**HSR (m)**	*Win*	33.9 ± 19.8	58.5
	*Loss*	16.1 ± 6.3	39.1		*Loss*	20.6 ± 10.1	49.2		*Loss*	34.0 ± 20.4	60.0

**ACC (count)**	*Win*	3.3 ± 1.2	38.2	**ACC (count)**	*Win*	4.0 ± 1.5	37.9	**ACC (count)**	*Win*	8.2 ± 3.4	41.6
	*Loss*	3.6 ± 1.2	35.3		*Loss*	4.4 ± 1.7	39.2		*Loss*	8.7 ± 3.3	38.8

**DEC (count)**	*Win*	2.0 ± 0.8	44.0	**DEC (count)**	*Win*	2.4 ± 1.0	43.2	**DEC (count)**	*Win*	4.3 ± 2.1	48.5
	*Loss*	2.0 ± 0.9	47.0		*Loss*	2.5 ± 1.3	53.5		*Loss*	4.3 ± 2.4	55.6

*Abbreviations:* TD = total distance, PL = PlayerLoad, AU = arbitrary units, S-W = standing-walking, JOG = jogging, RUN = running, HSR = high-speed running, ACC = accelerations, DEC = decelerations.

**TABLE 3:** Descriptive statistics for external peak demand variables according to quarter result across different time windows.

30-s time window				1-min time window				5-min time window			

*Variable*	*Result*	*Mean ± SD*	*%CV*	*Variable*	*Result*	*Mean ± SD*	*%CV*	*Variable*	*Result*	*Mean ± SD*	*%CV*
**TD (m)**	*Win*	80.4 ± 8.9	11.1	**TD (m)**	*Win*	134.0 ± 15.2	11.3	**TD (m)**	*Win*	454.9 ± 68.9	15.1
	*Tie*	81.4 ± 7.3	8.9		*Tie*	138.1 ± 10.8	7.8		*Tie*	458.5 ± 87.5	19.0
	*Loss*	78.3 ± 7.9	10.1		*Loss*	130.4 ± 13.3	10.2		*Loss*	433.9 ± 63.7	14.7

**PL (AU)**	*Win*	10.5 ± 1.5	14.9	**PL (AU)**	*Win*	17.1 ± 2.8	16.4	**PL (AU)**	*Win*	54.1 ± 10.9	20.1
	*Tie*	10.7 ± 1.3	12.5		*Tie*	17.2 ± 1.9	11.4		*Tie*	53.7 ± 11.6	21.5
	*Loss*	10.0 ± 1.5	14.9		*Loss*	16.5 ± 2.5	15.2		*Loss*	50.5 ± 10.2	20.2

**S-W (m)**	*Win*	33.0 ± 6.1	18.5	**S-W (m)**	*Win*	57.1 ± 13.8	24.3	**S-W (m)**	*Win*	216.7 ± 66.7	30.7
	*Tie*	33.4 ± 5.7	17.2		*Tie*	56.7 ± 12.4	21.9		*Tie*	222.7 ± 76.3	34.2
	*Loss*	33.1 ± 6.3	19.2		*Loss*	57.4 ± 15.1	26.4		*Loss*	213.4 ± 71.4	33.4

**JOG (m)**	*Win*	44.5 ± 10.0	22.6	**JOG (m)**	*Win*	70.4 ± 21.2	30.2	**JOG (m)**	*Win*	202.3 ± 107.6	53.1
	*Tie*	47.6 ± 8.2	17.3		*Tie*	72.9 ± 12.1	16.6		*Tie*	193.7 ± 50.4	26.0
	*Loss*	43.0 ± 8.4	19.6		*Loss*	67.7 ± 17.2	25.4		*Loss*	188.8 ± 95.1	50.3

**RUN (m)**	*Win*	26.6 ± 9.7	36.6	**RUN (m)**	*Win*	36.2 ± 18.5	51.2	**RUN (m)**	*Win*	82.8 ± 84.2	101.6
	*Tie*	24.4 ± 7.8	32.0		*Tie*	33.3 ± 10.6	31.9		*Tie*	69.7 ± 20.5	29.4
	*Loss*	25.8 ± 6.8	26.3		*Loss*	33.6 ± 9.5	28.4		*Loss*	71.1 ± 23.1	32.5

**HSR (m)**	*Win*	17.8 ± 8.7	48.9	**HSR (m)**	*Win*	20.9 ± 10.8	51.8	**HSR (m)**	*Win*	35.8 ± 20.8	58.3
	*Tie*	17.9 ± 8.5	47.4		*Tie*	20.5 ± 10.1	49.4		*Tie*	29.6 ± 16.2	54.6
	*Loss*	15.4 ± 7.0	45.8		*Loss*	17.4 ± 8.4	48.2		*Loss*	30.0 ± 17.3	57.7

**ACC (count)**	*Win*	3.3 ± 1.3	39.3	**ACC (count)**	*Win*	4.1 ± 1.6	34.6	**ACC (count)**	*Win*	8.3 ± 3.6	43.1
	*Tie*	3.0 ± 0.9	32.0		*Tie*	4.0 ± 1.2	39.9		*Tie*	8.3 ± 2.5	30.6
	*Loss*	3.4 ± 1.2	36.2		*Loss*	4.1 ± 1.4	34.6		*Loss*	8.0 ± 3.1	38.5

**DEC (count)**	*Win*	2.1 ± 0.9	42.8	**DEC (count)**	*Win*	2.5 ± 1.0	43.4	**DEC (count)**	*Win*	4.5 ± 2.1	47.1
	*Tie*	1.8 ± 0.9	51.0		*Tie*	2.5 ± 0.8	34.0		*Tie*	4.5 ± 1.9	42.6
	*Loss*	1.8 ± 0.8	46.2		*Loss*	2.2 ± 1.0	48.2		*Loss*	3.9 ± 2.1	55.6

*Abbreviations:* TD = total distance, PL = PlayerLoad, AU = arbitrary units, S-W = wtanding-walking, JOG = jogging, RUN = running, HSR = high-speed running, ACC = accelerations, DEC = decelerations.

**TABLE 4:** Descriptive statistics for external peak demand variables according to quarter point difference across different time windows.

30-s time window				1-min time window				5-min time window			

*Variable*	*Result*	*Mean ± SD*	*%CV*	*Variable*	*Result*	*Mean ± SD*	*%CV*	*Variable*	*Result*	*Mean ± SD*	*%CV*
**TD (m)**	*Low*	78.5 ± 7.7	9.81	**TD (m)**	*Low*	131.6 ± 12.8	9.7	**TD (m)**	*Low*	441.8 ± 70.1	15.8
	*High*	80.6 ± 9.0	11.21		*High*	134.1 ± 15.4	11.5		*High*	453.4 ± 68.2	15.0

**PL**	*Low*	10.1 ± 1.5	14.95	**PL**	*Low*	16.6 ± 2.4	14.8	**PL**	*Low*	51.5 ± 11.1	21.5
	*High*	10.6 ± 1.5	14.79		*High*	17.1 ± 2.8	16.4		*High*	53.9 ± 10.6	19.6

**S-W (m)**	*Low*	33.2 ± 6.3	19.11	**S-W (m)**	*Low*	57.9 ± 15.1	26.0	**S-W (m)**	*Low*	218.1 ± 73.1	33.5
	*High*	33.0 ± 6.0	18.35		*High*	56.7 ± 13.6	23.9		*High*	215.1 ± 65.7	30.5

**JOG (m)**	*Low*	43.6 ± 8.2	18.76	**JOG (m)**	*Low*	68.5 ± 16.1	23.5	**JOG (m)**	*Low*	190.7 ± 86.8	45.5
	*High*	44.6 ± 10.2	23.02		*High*	70.4 ± 21.6	30.6		*High*	202.2 ± 109.3	54.0

**RUN (m)**	*Low*	25.5 ± 6.8	26.84	**RUN (m)**	*Low*	33.8 ± 9.8	29.1	**RUN (m)**	*Low*	71.8 ± 23.7	32.9
	*High*	26.7 ± 9.8	36.98		*High*	36.2 ± 18.7	51.8		*High*	82.8 ± 85.6	103.4

**HSR (m)**	*Low*	16.1 ± 7.3	45.26	**HSR (m)**	*Low*	18.2 ± 8.6	47.4	**HSR (m)**	*Low*	30.6 ± 17.3	56.6
	*High*	17.7 ± 8.8	49.64		*High*	20.9 ± 11.0	52.6		*High*	35.7 ± 20.9	58.6

**ACC (count)**	*Low*	3.3 ± 1.2	36.04	**ACC (count)**	*Low*	4.1 ± 1.4	33.6	**ACC (count)**	*Low*	8.1 ± 3.0	37.8
	*High*	3.3 ± 1.3	39.38		*High*	4.1 ± 1.6	40.3		*High*	8.3 ± 3.5	43.0

**DEC (count)**	*Low*	1.8 ± 0.8	44.28	**DEC (count)**	*Low*	2.2 ± 1.0	44.8	**DEC (count)**	*Low*	4.0 ± 2.0	52.1
	*High*	2.1 ± 0.9	43.76		*High*	2.5 ± 1.1	43.9		*High*	4.5 ± 2.1	47.3

*Abbreviations:* TD = Total Distance, PL = Player Load, S-W = Standing-Walking, JOG = Jogging, RUN = running, HSR = High Speed Running, ACC = Accelerations, DEC = Decelerations.

Regarding game result, non-significant (p > 0.05), trivial to small differences were apparent in all variables across each time window between games that were won and lost. Differences in variables between games won and lost were mostly trivial in magnitude, with some variables reaching small effects in favor of wins (TD 30 s, PL 30 s, JOG 30 s, TD 1 min, S-W 1 min -0.28, JOG 1 min, and PL 5 min) or losses (ACC 30 s and ACC 1 min).

Concerning quarter result, significantly higher HSR (p = 0.03; ES = small) across the 1-min time window and PL (p = 0.04; ES = small) across the 5-min time window were evident during quarters that were won compared to lost. Non-significant (p > 0.05) differences were apparent in the remaining external PD variables across each time window. Differences according to quarter result (win vs. loss, win vs. tie, and tie vs. loss) were mostly trivial in magnitude, reaching small effects in favor of wins (vs. losses: TD 30 s, PL 30 s, HSR 30 s, DEC 30 s, TD 1 min, PL 1 min, HSR 1 min, DEC 1 min, TD 5 min, PL 5 min, HSR 5 min, and DEC 5 min; vs. ties: JOG 30 s, RUN 30 s, ACC 30 s, DEC 30 s, TD 1 min, and HSR 5 min) or ties (vs. losses: TD 30 s, PL 30 s, JOG 30 s, RUN 30 s, HSR 30 s, ACC 30 s, TD 1 min, PL min, JOG 1 min, HSR 1 min, DEC 1 min, TD 5 min, PL 5 min, and DEC 5 min).

Regarding quarter score-line, significantly greater PL (p = 0.03; ES = small) and DEC (p = 0.02; ES = small) across the 30-s time window, HSR (p = 0.04; ES = small) across the 1-min time window, and HSR (p = 0.04; ES = small) and DEC (p = 0.03; ES = small) across the 5-min time window were evident in quarters with high point differences between teams than quarters with low point differences. Non-significant (p > 0.05) differences were apparent in the remaining variables across each time window with mostly trivial effects and reaching small effects in favor of quarters with high point differences in some variables (TD 30 s, PL 30 s, DEC 30 s, HSR 1 min, DEC 1 min, PL 5 min, HSR 5 min, and DEC 5 min).

## DISCUSSION

The aim of this study was to quantify and compare the external PD encountered during games according to game result (win vs. loss), quarter result (win vs. tie vs. loss), and quarter point difference (± difference in score) in U18, male basketball players. The outcomes indicated that: (1) external PD does not clearly differentiate team success based on game result; (2) quarters that were won or tied showed higher external PD (small effects) compared to quarters that were lost; and (3) high point differences in quarter score-lines elicited higher external PD (small effects) than quarters with low point differences.

Regarding game result, non-significant (p > 0.05) differences with predominantly trivial effects were apparent across variables in each time window between games that were won and lost. Our findings contrast those made previously in basketball research exploring average external load intensities across entire games among national-level, U18, male basketball players [14]. Specifically, previous data show U18, male basketball players in lower-ranked teams (placed fifth to eighth) completed significantly (p < 0.05) higher relative distances (m · min^−1^) than players in top-ranked teams (placed first to fourth) [14]. In turn, data provided for adult, semi-professional, male basketball players show they performed significantly (p < 0.001) more high-intensity accelerations per minute during games that were lost compared to won [15]. These previous data suggest that less successful teams may exert higher average intensities across games, which may be due to tactical inefficiencies in movements (i.e., they do not move effectively across the court and thus perform more activity to execute a specific movement phase) or perform at greater intensities in an attempt to reduce the deficit in score-line when in a losing position rather than slowing play to protect a lead [13]. In contrast to previous findings [13, 14], our data suggest when external PD are quantified across shorter epochs (30-s, 1-min, and 5-min windows) rather than averaged across entire games, similar intensities are reached regardless of game result among U18, male players. The consistent external PD across shorter epochs in our study may be explained by the chaotic nature of basketball producing comparable intense periods of play for winning and losing teams, and knowing that determinants of success are multi-factorial [31]. In this regard, there are several offensive and defensive technical-tactical performance indicators that have been shown to influence game outcome among various basketball player samples including rebounds [31], passing skills (e.g. ball coordination between inside and outside playing positions that enhance threepoint shot opportunities) [32], perceptive and decision-making processes [33], and defensive preparation (e.g. personal fouls, steals) [34]. In turn, our data suggest identifying factors associated with team success in U18, male basketball players is not permissible using PD variables gathered from external load monitoring data in isolation [35], with a multidimensional approach considering other areas of performance likely needed.

Similar to what was found across entire games, quarter analyses showed non-significant differences in external PD across all variables and time windows except HSR across a 1-min window and PL across a 5-min window (higher values in quarters that were won). These findings contrast previous data provided for adult, semi-professional, male basketball players showing higher (p > 0.05, small effects) external PD for PL · min^−1^ (4-min and 5-min windows) during quarters that were lost compared to won [13]. Previously, Fox et al. [13] postulated that the higher peak PL · min^−1^ during quarters that were lost was potentially due to the team adopting an increased playing pace to maximize scoring opportunities and reduce the score-line margin [13]. In turn, the team analyzed in our study were not typically in a losing position (won 8 out of 9 games) and may have performed at high external intensities during game quarters that were won to establish the lead but reduced movement intensities in subsequent quarters (that were lost) to protect the lead or due to diminished motivation and concentration if the game result was clearly decided. In support of this notion, past research examining adult, professional, male basketball players indicates a greater difference in score-line at the beginning of each game quarter corresponds with a higher number of recovered points by the losing team in that quarter [17]. Further, the authors [17] postulated that players in a losing position may increase their work rate to score more points and heighten their defensive readiness, which may both exacerbate the external PD performed. Although this previous research was provided in adult, male basketball players, our data indicate similar scenarios may occur in U18, male basketball players; however, further research is encouraged exploring the motivational factors in young basketball players during different game scenarios to confirm this supposition.

A unique aspect of this study was the deeper analysis of quarter results through considering the score-line margin when examining external PD. In this regard, although greater external PD were attained (PL 30 s, HSR 30 s, 1 min and 5 min, and DEC 5 min) with high point differences than low point differences in score-line, the differences were small in magnitude. Nevertheless, these small differences support the notion that the U18, male basketball players in the examined team may have performed at higher external intensities across quarters to establish score-line leads involving high margins, but then reduced movement intensities given they were in a strong winning position yielding quarters with low score-line margins. Furthermore, the higher external PD for HSR across all time windows during quarters with high score-line differences may indicate fast break scenarios or elevated pace in transitions (given speeds > 18 km · h^−1^ had to be reached for HSR to be detected) across the court can be key tactical strategies that promote pronounced leads in the score-line.

The limitations encountered in carrying out this study should be considered when interpreting our results. First, only external load variables were monitored, therefore internal PD (e.g., heart rate, rating of perceived exertion) were not explored and may show different patterns to those observed in our study for external PD. Second, due to the real-world context our observational analyses were conducted in, the reported outcomes were bound by the results of the included games and therefore games involving score-line margins outside of those seen in the present study may induce different trends in PD according to game and quarter results. Furthermore, the team won most games (8 out of 9 games) and therefore the data points were not distributed in a balanced manner for all factors investigated (game result, quarter result, and quarter point difference). Third, the sample size adopted in this study was small given a single basketball team was recruited, with the sample size for lost games only representative of a single game. Consequently, these findings may not translate to all basketball teams given the variations in success, tactical strategies, playing pace, and player fitness likely evident within and between competitions.

## CONCLUSIONS

External PD appear to remain consistent regardless of game result in U18, male basketball players. In turn, although quarters that were won and involving high score-line margins elicited higher external PD than quarters that were lost or close in score-line, the observed effects were only trivial to small in magnitude. Accordingly, the external PD attained during games may not be a key indicator of overall team success in games. Further analyses using a wider range of variables in other areas such as technical-tactical parameters, psychology, team strategies, competition level, and player experience are recommended to identify the determinants of team success in young basketball players.

## References

[1] Svilar L, Castellano J, Jukic I. Comparison of 5vs5 Training Games and Match Play using Microsensor Technology in Elite Basketball. J Strength Cond Res. 2019; 33(7):1897–903. 30204654 10.1519/JSC.0000000000002826

[2] O’grady CJ, Dalbo VJ, Teramoto M, Fox JL, Scanlan AT. External Workload can be Anticipated During 5 vs. 5 Games-Based Drills in Basketball Players: An Exploratory Study. Int J Environ Res Public Health. 2020; 17(6):2103. 32235721 10.3390/ijerph17062103 PMC7143829

[3] Sosa C, Lorenzo A, Trapero J, Ribas C, Alonso E, Jimenez SL. Specific Absolute Velocity Thresholds during Male Basketball Games Using Local Positional System; Differences between Age Categories. Appl Sci. 2021; 11(10):4390.

[4] Aughey RJ, Falloon C. Real-time Versus Post-Game GPS Data in Team sports. J Sci Med Sport. 2010; 13(3):348–9. 19589726 10.1016/j.jsams.2009.01.006

[5] Impellizzeri F, Marcora S, Coutts A. Internal and External Training Load: 15 Years On. Int J Sports Physiol Perform. 2018; 14(2):270–3. 10.1123/ijspp.2018-0935 30614348

[6] Jayanthi N, Schley S, Cumming SP, Myer GD, Saffel H, Hartwig T, et al. Developmental Training Model for the Sport Specialized Youth Athlete: A Dynamic Strategy for Individualizing Load-Response During Maturation. Sports Health. 2022; 14(1):142–53. 34763556 10.1177/19417381211056088 PMC8669935

[7] Abdelkrim BN, El Fazaa S, El Ati J. Time-Motion Analysis and Physiological Data of Elite Under-19-Year-Old Basketball Players During Competition. Br J Sports Med. 2007; 41(2):69–75. 17138630 10.1136/bjsm.2006.032318 PMC2658931

[8] Scanlan AT, Dascombe BJ, Reaburn P, Dalbo VJ. The Physiological and Activity Demands Experienced by Australian Female Basketball Players During Competition. J Sci Med Sport. 2012 Jul; 15(4):341–7. 22244965 10.1016/j.jsams.2011.12.008

[9] Vázquez-Guerrero J, Vizuete JJ, Garcia F, Hughes J, De Ste Croix MBA, Ayala F. The most demanding scenarios of 5-on-5 modified scrimmage situations in elite basketball. J Strength Cond Res. 2020 Apr 1; 17(1):1–11. 10.23736/S0022-4707.21.11613-5 33528216

[10] Alonso Pérez-Chao E, Gómez MÁ, Lisboa P, Trapero J, Jiménez SL, Lorenzo A. Fluctuations in External Peak Demands across Quarters during Basketball Games. Front Physiol. 2022;13:868009. doi: 10.3389/fphys.2022.868009. 35492582 PMC9039040

[11] Alonso Perez-Chao E, Lorenzo A, Scanlan A, Lisboa P, Sosa C, Gómez MA. Higher Playing Times Accumulated Across Entire Games and Prior to Intense Passages Reduce the Peak Demands Reached by Elite, Junior, Male Basketball Players. Am J Mens Health. 2021. “in press”. 10.1177/15579883211054353 PMC8558607 34720014

[12] Alonso E, Miranda N, Zhang S, Sosa C, Trapero J, Lorenzo J, et al. Peak Match Demands in Young Basketball Players: Approach and Applications. Int J Environ Res Public Health. 2020; 17(7):2256. 32230798 10.3390/ijerph17072256 PMC7177956

[13] Fox J, Jeese G, Scanlan A. Not All about the Effort? A Comparison of Playing Intensities During Winning and Losing Game Quarters in Basketball. Int J Sports Physiol Perform. 2021; 16(9):1378–81. 33662929 10.1123/ijspp.2020-0448

[14] Pino-Ortega J, Rojas-Valverde D, Gómez-Carmona CD, Bastida-Castillo A, Hernández-Belmonte A, García-Rubio J, et al. Impact of contextual factors on external load during a congested-fixture tournament in elite U’18 basketball players. Front Psychol. 2019; 10:1100. 31156514 10.3389/fpsyg.2019.01100 PMC6529817

[15] Fox J, Stanton R, Sargent C, O’Grady C, Scanlan A. The Impact of Contextual Factors on Game Demands in Starting, Semiprofessional, Male Basketball Players. Int J Sports Physiol Perform. 2020; 15(4):450–6. 10.1123/ijspp.2019-0203 31605525

[16] Gómez MÁ, Silva R, Lorenzo A, Kreivyte R, Sampaio J. Exploring the Effects of Substituting Basketball Players in High-level Teams. J Sports Sci. 2017; 35(3):247–54. 26986448 10.1080/02640414.2016.1161217

[17] Sampaio J, Lago C, Casais L, Leite N. Effects of Starting Score-line, Game Location, and Quality of Opposition in Basketball Quarter Score. Eur J Sport Sci. 2010; 10(6):391–6.

[18] García F, Castellano J, Reche X, Vázquez-Guerrero J. Average Game Physical Demands and the Most Demanding Scenarios of Basketball Competition in Various Age Groups. J Hum Kinet. 2021; 79(1):165–74. 34400996 10.2478/hukin-2021-0070 PMC8336539

[19] Harriss DJ, Atkinson G. Ethical Standards in Sport and Exercise Science Research: 2014 Update. Int J Sport Med. 2014; 34:1025–9. 10.1055/s-0033-1358756 24293054

[20] Hodder RW, Ball KA, Serpiello FR. Criterion Validity of Catapult Clearsky T6 Local Positioning System for Measuring Inter-unit Distance. Sensors. 2020; 20(13):3693. 32630274 10.3390/s20133693 PMC7374308

[21] Luteberget L, Spencer M, Gilgien M. Validity of the Catapult ClearSky T6 Local Positioning System for Team Sports Specific Drills, in Indoor Conditions. Front Physiol. 2018; 9:115. 29670530 10.3389/fphys.2018.00115 PMC5893723

[22] Serpiello FR, Hopkins WG, Barnes S, Tavrou J, Duthie GM, Aughey RJ, et al. Validity of an Ultra-Wideband Local Positioning System to Measure Locomotion in Indoor Sports. J Sports Sci. 2018; 36(15):1727–33. 29192842 10.1080/02640414.2017.1411867

[23] Hoppe MW, Baumgart C, Polglaze T, Freiwald J. Validity and Reliability of GPS and LPS for Measuring Distances Covered and Sprint Mechanical Properties in Team Sports. PLoS One. 2018; 13(2):e0192708. 29420620 10.1371/journal.pone.0192708 PMC5805339

[24] Luteberget L, Holme B, Spencer M. Reliability of Wearable Inertial Measurement Units to Measure Physical Activity in Team Handball. Int J Sports Physiol Perform. 2017; 13(4):467–73. 10.1123/ijspp.2017-0036 28872371

[25] Castellano J, Casamichana D, Calleja-González J, Román JS, Ostojic S. Reliability and Accuracy of 10 Hz GPS Devices for Short-Distance Exercise. J Sport Sci Med. 2011; 10(1):233–4. PMC3737891 24137056

[26] Johnston RJ, Watsford ML, Kelly SJ, Pine MJ, Spurrs RW. Validity and Interunit Reliability of 10 hz and 15 hz GPS Units for Assessing Athlete Movement Demands. J Strength Cond Res. 2014; 28(6):1649–55. 24276300 10.1519/JSC.0000000000000323

[27] Cunningham DJ, Shearer DA, Carter N, Drawer S, Pollard B, Bennett M, et al. Assessing Worst Case Scenarios in Movement Demands Derived from Global Positioning Systems During International Rugby Union Matches: Rolling Averages Versus Fixed Length Epochs. PLoS One. 2018; 13(4):1–14. 10.1371/journal.pone.0195197 PMC5886488 29621279

[28] García F, Schelling X, Castellano J, Martín-García A, Pla F, Vázquez-Guerrero J. Comparison of the most demanding scenarios during different in-season training sessions and official matches in professional basketball players. Biol Sport. 2022; 237–44. 35309543 10.5114/biolsport.2022.104064 PMC8919871

[29] Svilar L, Castellano J, Jukic I. Load monitoring system in top-level basketball team: Relationship between external and internal training load. Kinesiology. 2018; 50(1):25–33.

[30] Hopkins W, Marshall S, Batterham A, Hanin J. Progressive Statistics for Studies in Sports Medicine and Exercise Science. Med Sci Sports Exerc. 2009; 41(1):3–12. 19092709 10.1249/MSS.0b013e31818cb278

[31] Zhang S, Gomez MÁ, Yi Q, Dong R, Leicht A, Lorenzo A. Modelling the Relationship between Match Outcome and Match Performances during the 2019 FIBA Basketball World Cup: A Quantile Regression Analysis. Int J Environ Res Public Health. 2020; 17(16):5722. 32784740 10.3390/ijerph17165722 PMC7460061

[32] Zhang S, Lorenzo A, Woods CT, Leicht AS, Gómez MA. Evolution of Game-play Characteristics within-season for the National Basketball Association. Int J Sport Sci Coach. 2019; 14(3):355–62.

[33] Lorenzo A, Gómez MÁ, Ortega E, Ibáñez SJ, Sampaio J. Game Related Statistics which Discriminate between Winning and Losing Under-16 Male Basketball Games. J Sport Sci Med. 2010; 9(4):664–8. PMC3761811 24149794

[34] Castillo D, Raya-González J, Clemente FM, Conte D, Rodríguez-Fernández A. The Effects of Defensive Style and Final Game Outcome on the External Training Load of Professional Basketball Players. Biol Sport. 2021; 38(3):483–90. 34475630 10.5114/biolsport.2021.101124 PMC8329980

[35] Hughes M, Bartlett R. The Use of Performance Indicators in Performance Analysis. J Sports Sci. 2002; 20(10):739–54. 12363292 10.1080/026404102320675602

